# The timing of minimally invasive surgery for prenatally diagnosed choledochal cysts

**DOI:** 10.1186/s12887-024-04734-x

**Published:** 2024-04-11

**Authors:** Shiwen Pan, Wei Li, Huan Chen, Changgui Lu

**Affiliations:** 1 Department of Anesthesia and Operation, Children’s Hospital of Nanjing Medical University, Nanjing, 210008 China; 2 Department of Neonatal Surgery, Children’s Hospital of Nanjing Medical University, 72 Guangzhou Road, Nanjing, 210008 China

**Keywords:** Newborn, Young infant, Choledochal cyst, Timing of surgery, Minimally invasive

## Abstract

**Objective:**

There are no clear evidence-based recommendations concerning when patients with prenatally diagnosed choledochal cysts (CCs) should undergo surgery. This study was primarily designed to explore the proper timing of minimally invasive surgery for prenatally diagnosed CC patients.

**Methods:**

Seventy-three patients with prenatally diagnosed CC were enrolled in this study and divided into 4 subgroups according to age at surgery (15 patients in the < 1 month group, 27 in the 1–2 months group, 14 in the 2–3 months group and 17 in the > 3 months group). Eighty-five healthy infants were recruited and divided into 4 age groups (29 in the < 1 month group, 20 in the 1–2 month group, 19 in the 2–3 month group and 17 in the > 3 month group). Preoperative data were collected and compared between CC patients and healthy controls in 4 age groups. Additionally, 73 patients were divided into laparoscopic and open groups to compare postoperative recovery indices and the occurrence of complications to determine the safety and feasibility of laparoscopic CC application in neonates and young infants.

**Results:**

Twenty-one of 73 (28.8%) patients who were prenatally diagnosed with CCs experienced various clinical symptoms, and 15 of 21 (71.4%) patients experienced clinical symptoms less than 2 months after birth. No differences were found in alanine transaminase (ALT), aspartate transaminase (AST) or aspartate transaminase (APRI) levels between CC patients and controls at ≤ 1 month or 1–2 months of age (all *p* > 0.05), while higher levels were found in CC patients at 2–3 months or > 3 months of age (all *p* < 0.05). ALT, AST and DBIL levels 1 week after surgery were significantly lower than those before surgery in CC patients who underwent laparoscopic CC excision at > 2 months of age, while DBIL levels 1 week after surgery were also significantly lower than those before surgery in patients who underwent CC excision at ≤ 2 months of age. The initial oral feeding time in the laparoscopic surgery group was significantly earlier than that in the open surgery group for both CC patients who underwent CC excision at ≤ 2 months of age and those > 2 months of age (all *p* < 0.05). No differences were found in the rates of anastomotic leakage or stricture formation between the laparoscopic and open surgery groups at ≤ 2 months or > 2 months of age.

**Conclusion:**

Most clinical symptoms attributed to CC occur less than 2 months after birth, while liver function and liver fibrosis may deteriorate after 2 months of age in patients with prenatally diagnosed CC. Laparoscopic surgery for CC in newborns and young infants (either less than or more than 2 months old) is safe and feasible and can shorten the initial oral feeding time without increasing complications such as postoperative anastomotic leakage or stricture. Thus, performing laparoscopic CC excisions within 2 months after birth in patients with prenatally diagnosed CC may be appropriate.

## Introduction

Choledochal cyst (CC) or congenital biliary dilation is a rare malformation that has a skewed distribution with hereditary features and is far more common in East Asian females [1] than in males. Early diagnosis and treatment are usually needed for CC since there are risks for malignancy in the bile ducts and biliary obstruction [2], which may result in liver fibrosis with age. Laparoscopic cyst resection followed by Roux-en-Y hepaticojejunostomy is still the most effective and radical surgical procedure for correcting this disorder. Currently, with improvements in access to prenatal ultrasound examination, nearly 70% of CCs can be identified during the prenatal period [3, 4]; however, most prenatally diagnosed CCs are asymptomatic during the neonatal or young infant period. Due to the limited abdominal space available for laparoscopic surgery and the weak tolerance of long-term general anesthesia [5, 6], radical procedures are usually postponed to 3–6 months of age [7]. According to a previous report [8], delaying radical surgery in asymptomatic patients up to a weight of more than 5.6 kg is associated with a decrease in postoperative complications. However, several reports [9–12] have suggested that CC could result in liver fibrosis in young infants, even during the neonatal period. Furthermore, delaying surgery to 3–6 months after birth also increases the risk of CC symptoms, and 24%∼62% of symptoms occur within the first 3 months after birth [3, 8, 13]. It has been proven that surgery after symptoms appear is accompanied by relatively greater rates of late complications, such as anastomotic stricture and cholangitis, than surgery before symptoms occur [3]. Determining the proper timing of minimally invasive surgery for prenatally diagnosed choledochal cysts is still an important issue for pediatric patients.

This study was primarily designed to explore the proper surgical timing for prenatally diagnosed CC patients. The feasibility and safety of minimally invasive surgery in young infants were also evaluated.

## Methods

### Patients and controls

A retrospective survey was conducted on patients with CC who underwent surgical CC excision between January 2013 and December 2022 at the Children’s Hospital of Nanjing Medical University. The inclusion criteria were as follows: ① patients who were diagnosed with CC prenatally, ② whose diagnosis was confirmed during the operation, and ③ whose age at CC excision was less than 6 months. The exclusion criteria were as follows: ① patients who underwent CC without surgery, ② patients who underwent staging surgery, and ③ patients who were lost to follow-up. A total of 85 relatively healthy infants (admitted to the hospital for indirect inguinal hernia, lymphangioma, epidermoid cyst or dermoid cyst on the body surface) aged 0–6 months were recruited as the control group. All CC patients finished 1–10 years of follow-up after CC excisions.

### Indications for CC excision

Patients with prenatally diagnosed CC are well followed up after birth, and CC excision can be performed as soon as possible for CC patients with clinical symptoms, such as vomiting, rapidly enlarged cysts, or clay-colored stool. For patients with cholestasis, defined as abnormal direct/conjugated bilirubin is defined as a serum value > 1.0 mg/dl according to the Joint Recommendations of the North American Society for Pediatrics [14], CC excision was also performed when cholestasis did not improve after 1–2 weeks of oral ursodeoxycholic acid treatment. For patients without any clinical symptoms, CC excisions were elective and recommended to be performed within 6 months after birth.

In our center, laparoscopic CC excisions have been used extensively for old infants or children with CCs since 2013; however, these methods became the standard protocols for neonates and young infants with CC until 2018. All CC patients underwent laparoscopic CC excision after October 2018. In the present study, young infants who were prenatally diagnosed with CC (aged less than 6 months), who underwent CC excision after October 2018, who underwent laparoscopic surgery were enrolled in the laparoscopic surgery group, and those who underwent CC excision earlier than October 2018 via open surgery were recruited into the open surgery group.

### Laparoscopic surgery

Under general anesthesia, a 5 mm trocar for a 5 mm 30° laparoscope was placed in the center of the umbilical region. Two additional 3 mm trocars were inserted at the right upper and lower abdomen, while one 3 mm trocar was placed at the left upper abdomen. After performing a cholangiogram by injecting contrast material from the gallbladder, the gallbladder was removed from the gallbladder bed, and the cyst was completely resected proximal to the common hepatic duct and distal to the pancreatic duct. Then, the jejunum 20 cm distal to the Treitz ligament was identified and exteriorized through the umbilical port site. An approximately 25 cm long Rouxen-Y limb was constructed without laparoscopy and then returned to the abdomen. After re-establishing the pneumoperitoneum, the Roux loop was delivered to the hilum via a retrocolic path. Finally, an end-to-side hepaticojejunostomy was performed via laparoscopy.

### Study design and statistical indicators

Patients with CC were divided into ≤ 1 month, 1–2 month, 2–3 month and > 3 month groups according to the age at surgery, while healthy infants were also divided into 4 age groups, the same as patients with CC. Sex, weight at surgery, preoperative alanine aminotransferase (ALT) level, preoperative aspartate aminotransferase (AST) level, cholestasis status and aspartate aminotransferase-to-platelet ratio index (APRI) were collected from CC patients and compared with those of healthy infants in various age groups to determine the proper timing of CC excision. The birth weight, gestational age, CC size, and clinical symptoms, such as cholestasis, vomiting, and clay-colored stool, of CC patients were also collected and evaluated. CC patients were also divided into laparoscopic surgery and open surgery groups ≤ 2 months and > 2 months old, respectively, to evaluate the feasibility and safety of performing minimally invasive surgery in young infants. ALT, AST, γ-glutamyl transpeptidase (γ-GT), direct bilirubin (DBIL) 1 week after surgery, initial oral feeding time after surgery, parenteral nutrition (PN) time, and postoperative hospital stay data were also collected and compared between patients who underwent laparoscopic surgery and those who underwent open surgery. CC size was defined as the average height, width and antero-posterior diameter on MRI.

$$\text{A}\text{P}\text{R}\text{I}=\text{A}\text{S}\text{T} \text{l}\text{e}\text{v}\text{e}\text{l}/\text{A}\text{S}\text{T}\left(\text{u}\text{p}\text{p}\text{e}\text{r} \text{l}\text{i}\text{m}\text{i}\text{t} \text{o}\text{f} \text{n}\text{o}\text{r}\text{m}\text{a}\text{l}\right)/\text{P}\text{L}\text{T} \text{c}\text{o}\text{u}\text{n}\text{t}\text{s}\left({10}^{9}\right)\times100$$

**Fig. 1:**
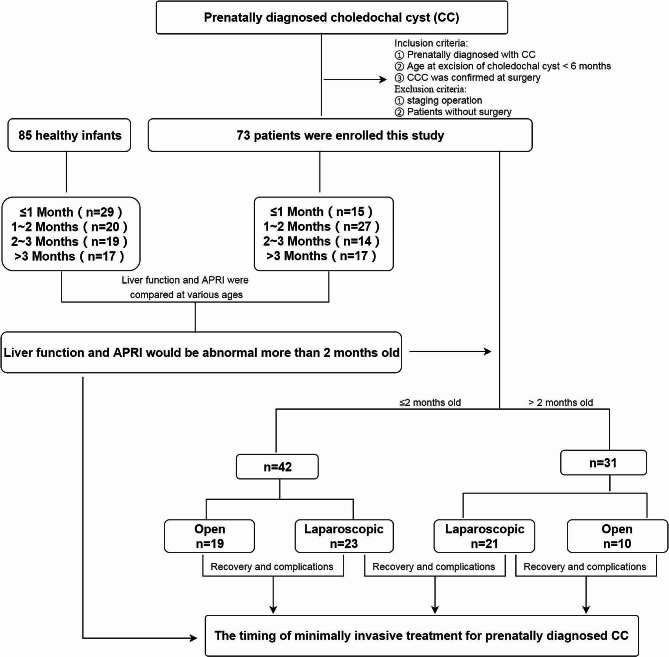
Flowchart of the study

This study was approved and supervised by the Institutional Ethics Committee of Nanjing Children’s Hospital of Nanjing Medical University (Approval No: 202402004-1), and the flow chart is shown in Fig. 1.

### Statistical analysis and software

The data are presented as percentages (%), medians (P25, P75), and means ± SDs, and the statistical significance was set at 0.05. The distribution of continuous variables was examined. A t test was applied for normally distributed data, and a rank-sum test was applied for nonnormally distributed data. Categorical variables were tested using the chi-square (χ2) test or Fisher’s exact test. SPSS software (version 22.0) was used for all the statistical analyses. In the present study, because of the nonnormal distribution of the data, a rank-sum test (Mann‒Whitney U test) was used. The Wilcoxon test was used to compare the differences in liver function between 1 week after surgery and before surgery.

## Results

### Clinical characteristics of patients with choledochal cysts and healthy control children at various ages

A total of 73 patients with CC (70 with Todani type I and 3 with Todani type II) and 85 controls were enrolled in this study and divided into 4 age groups: ≤1 month (CC, *n* = 15; control, *n* = 29), 1–2 months (CC, *n* = 27; control, *n* = 20), 2–3 months (CC, *n* = 14; control, *n* = 19) and > 3 months (CC, *n* = 17; control, *n* = 17). Up to January 2024, all CC patients were followed up for 1 ∼ 10 years, and no deaths occurred. Various clinical symptoms were found in 21/73 (28.8%) patients with prenatally diagnosed CC in this study, including 19 with cholestasis, 1 with clay-colored stool and 1 with a rapid increase in CC size. Among the 19 patients with cholestasis, 2 had clay-colored stool, 2 had vomiting, and 1 had a rapid increase in CC size. Importantly, 15/21 (71.4%) clinical symptoms occurred less than 2 months after birth. CC size in patients with clinical symptoms was significantly greater than that in patients without clinical symptoms (44.33 [35.50, 63.75] vs. 30.34 [22.47, 42.88] mm, *p* = 0.001); however, no difference in CC size was detected among CC patients of various ages (*p* > 0.05). No differences were found in gestational age, birth weight, or occurrence of clinical symptoms among patients with prenatally diagnosed CC at various ages (all *p* > 0.05). The details are shown in Table 1.

**Table 1:** Baseline data of patients who were prenatally diagnosed with CC at various ages

Age at surgery	≤ 1 month(*n* = 15)	1–2 months (*n* = 27)	2–3 months (*n* = 14)	> 3 months (*n* = 17)	P
Gestational age < 37 weeks(Yes/No, n)	1/14	2/25	0/14	0/17	0.599
Sex(Male/Female, n)	7/8	7/20	2/12	1/16	0.045
Birth weight (kg, M[P25,P75])	3.20[3.00, 3.50]	3.25[3.05, 3.40]	3.33[3.10, 3.40]	3.50[3.28, 3.73]	0.057
^a^Clinical symptoms(Yes/No, n)	8/7	7/20	3/11	3/14	0.146
^b^CC size (mm, M[P25,P75])	35.67[28.33, 46.67]	37.66[28.67,49.67]	29.0[19.0,35.25]	31.33[23.74, 66.25]	0.169

a: Clinical symptoms were identified in 21 patients, including 19 with cholestasis, 1 with clay-colored stool and 1 with a rapid increase in CC size. In 19 patients with cholestasis, 2 had clay-colored stool, 2 had vomiting, and 1 had a rapid increase in CC size. b: CC size in patients with clinical symptoms was significantly greater than that in patients without clinical symptoms (44.33 [35.50, 63.75] vs. 30.34 [22.47, 42.88] mm, *p* = 0.001); however, no difference in CC size was detected among CC patients of various ages. M means median

**Fig. 2:**
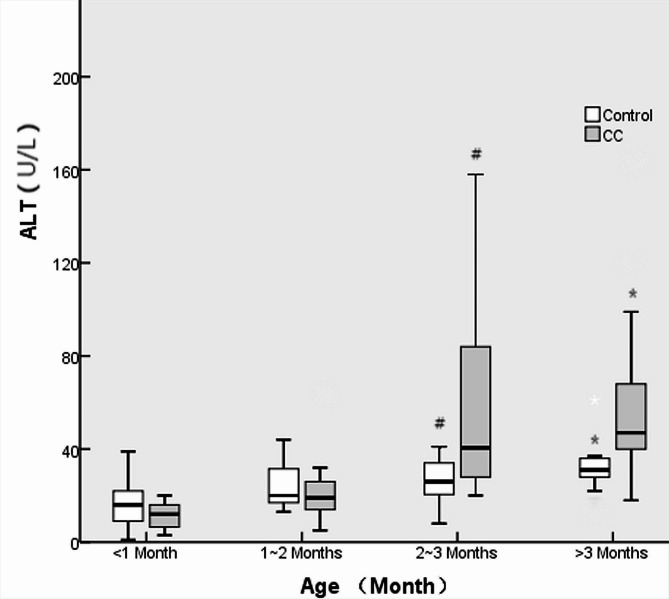
ALT levels in CC patients and controls at various ages. # and * indicate *p* < 0.05

**Fig. 3:**
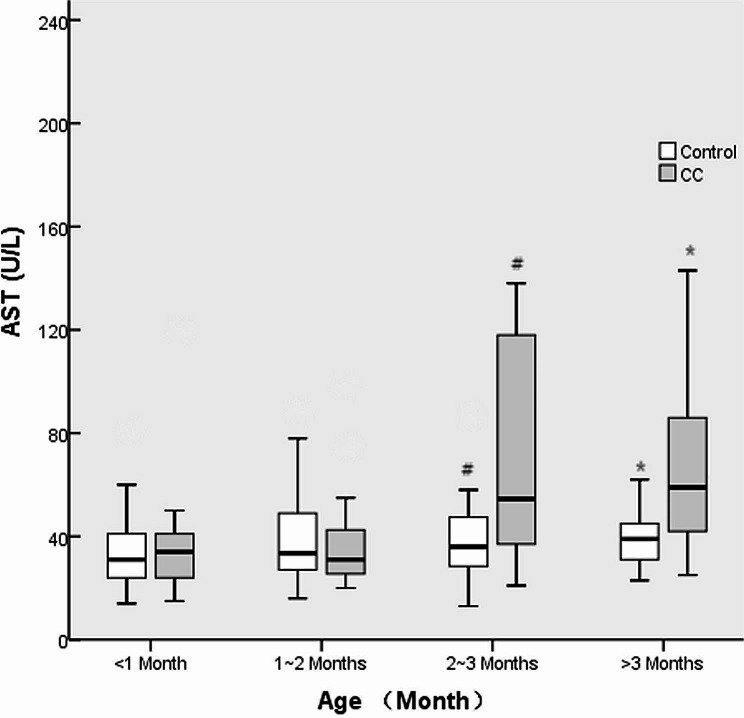
AST levels in CC patients and controls at various ages. # and * indicate *p* < 0.05

**Fig. 4:**
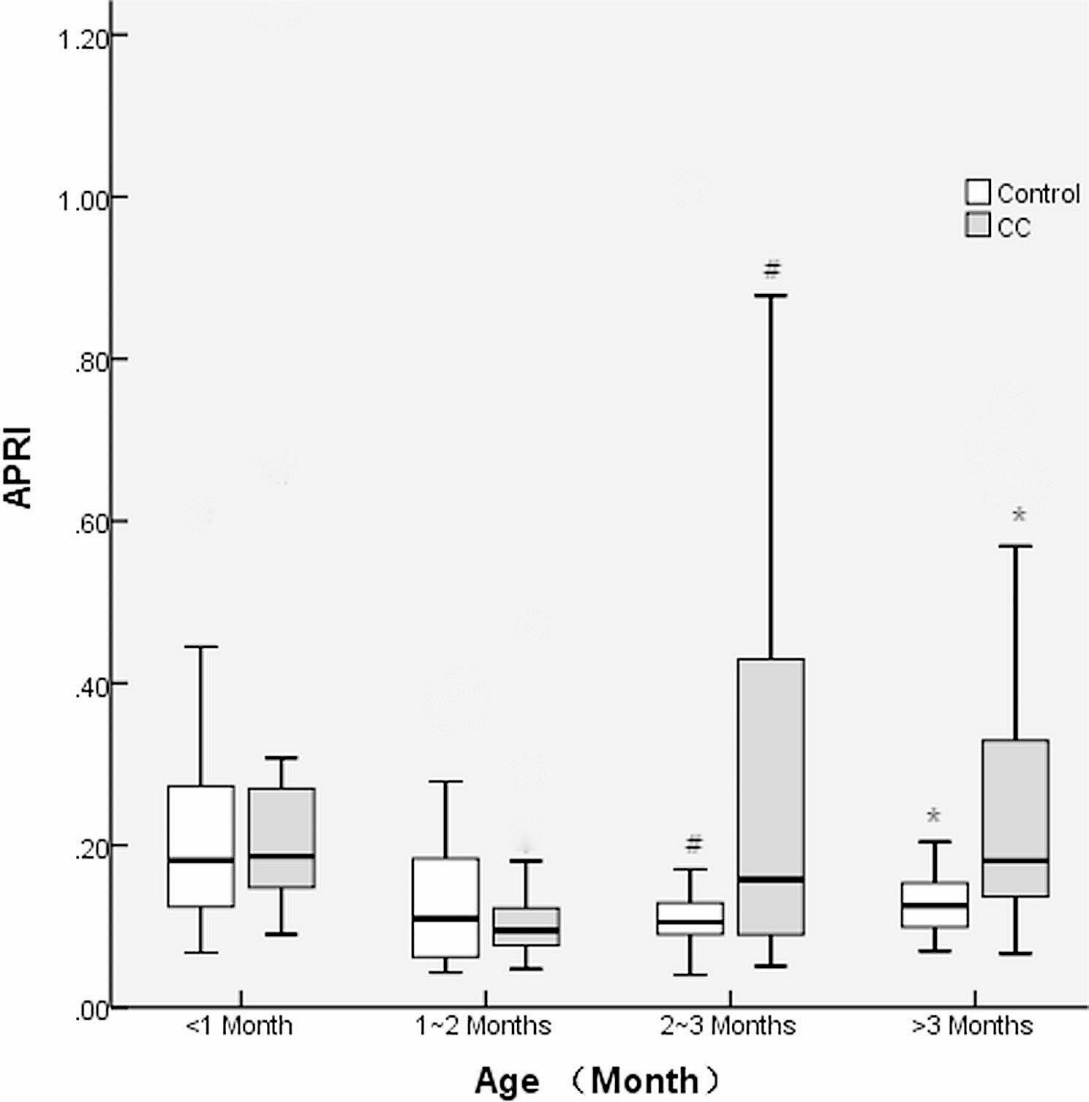
APRI of CC patients and controls at various ages. # and * indicate *p* < 0.05

There were no differences in the male/female ratio or weight between the CC and control groups at various ages (all *p* > 0.05). No differences were found in the ALT, AST or APRI between CC patients and controls at ≤ 1 month or 1–2 months (all *p* > 0.05), while higher levels were found in CC patients at 2–3 months or > 3 months (all *p* < 0.05). There were greater rates of cholestasis in CC patients than in controls at ≤ 1 month and 1–2 months of age (all *p* < 0.05); however, there was no significant difference in the incidence of cholestasis among all ages in CC patients (46.7% vs. 22.2% vs. 21.4% vs. 17.6%, *p* = 0.280). The details are shown in Table 2; Figs. 2, 3, and 4.

**Table 2:** Clinical characteristics of patients with choledochal cysts before surgery and healthy control children at various ages

Age at surgery and group		Sex (Male/Female, n)	Weight (kg, M[P25,P75])	ALT (U/L, M[P25,P75])	AST (U/L, M[P25,P75])	^a^Cholestasis (Yes/No, n)	APRI (M[P25,P75])
≤ 1 Month	CC (*n* = 15)	7/8	3.60[3.00,3.95]	12.00[5.00,16.00]	34.00[21.00,43.00]	7/8	0.30[0.19,0.90]
	Control (*n* = 29)	8/21	3.80[3.45,4.35]	16.00[9.00,12.00]	31.00[22.00,42.50]	1/28	0.32[0.18,0.45]
	p	0.206	0.056	0.104	0.921	0.002	0.465
1–2 Months	CC (*n* = 27)	7/20	4.50[4.00,4.80]	19.00[13.00,26.00]	31.00[25.00,44.00]	6/21	0.09[0.08,0.13]
	Control (*n* = 20)	4/16	4.75[4.03,5.20]	20.00[16.50,31.75]	33.50[27.00,49.00]	0/20	0.11[0.06,0.19]
	p	0.900	0.156	0.093	0.539	0.031	0.863
2–3 Months	CC (*n* = 14)	2/12	5.50[5.23,6.00]	40.50[27.00,90.75]	54.50[36.25,121.75]	3/11	0.16[0.09,0.44]
	Control (*n* = 19)	6/13	5.90[5.15,6.30]	26.00[18.00,34.00]	36.00[27.00,48.00]	0/19	0.11[0.09,0.13]
	p	0.416	0.265	0.013	0.035	0.067	0.045
> 3 Months	CC (*n* = 17)	1/16	7.00[6.35,7.90]	47.00[39.00,70.00]	59.00[42.00,87.50]	3/14	0.18[0.14,0.45]
	Control (*n* = 17)	2/15	7.10[6.25,7.90]	31.00[27.00,36.50]	39.00[30.50,45.00]	0/17	0.13[0.10,0.16]
	p	1.000	0.972	0.002	0.004	0.227	0.010

a: There were no differences in the incidence of cholestasis among the 4 age groups at surgery (46.7% vs. 22.2% vs. 21.4% vs. 17.6%, *p* = 0.280)

M indicates the median

### Clinical characteristics and recovery after CC excision in the laparoscopic surgery and open surgery groups at various ages

Seventy-three CC patients were divided into ≤ 2 months (*n* = 42) and > 2 months (*n* = 31) of age and further divided into a laparoscopic group (*n* = 23, ≤ 2 months of age and *n* = 21, > 2 months of age) and an open group at every age (*n* = 19, ≤ 2 months of age and *n* = 10, > 2 months of age). No patient in the laparoscopic group had to be converted to open surgery because of intraoperative difficulties or complications.

There were no differences in the male/female ratio, age at surgery, weight at surgery, or preoperative AST, γ-GT or DBIL levels between the laparoscopic and open surgery groups at ≤ 2 months and > 2 months of age (all *p* > 0.05). The preoperative ALT level was slightly greater in the laparoscopic group than in the open group at ≤ 2 months of age (*p* = 0.013), which was within the normal range. The details are shown in Table 3.

ALT, AST and DBIL levels 1 week after surgery were significantly lower than those before surgery in patients who underwent laparoscopic CC excision at > 2 months of age, while DBIL levels 1 week after surgery were also significantly lower than those before surgery in patients who underwent laparoscopic CC excision at ≤ 2 months of age. No differences were found in the γ-GT levels between 1 week after surgery and before surgery at ≤ 2 months or > 2 months of age in CC patients who underwent laparoscopic CC excision (all *p* > 0.05). The details are indicated in Table 4.

The operative time was significantly longer in the laparoscopic group than in the open group, either at ≤ 2 months of age or > 2 months of age (all *p* < 0.05). The initial oral feeding time in the laparoscopic surgery group was significantly earlier than that in the open surgery group, either at ≤ 2 months of age or > 2 months of age (all *p* < 0.05). No differences were found in the rates of anastomotic leakage or stricture formation between the laparoscopic and open surgery groups at ≤ 2 months or > 2 months of age. The PN duration and postoperative hospital stay were shorter in the laparoscopic group than in the open group at > 2 months of age. The details are shown in Table 5.

There were no differences in operative time, initial oral feeding time, PN time, postoperative hospital stay, or rates of anastomotic leakage or stricture between CC patients who underwent laparoscopic surgery at less than 2 months of age and those who were more than 2 months of age (all *p* > 0.05). The details are indicated in Table 6.

**Table 3:** Baseline data between the laparoscopic and open surgery groups at various ages

Age at surgery Group	≤ 2 Months (*n* = 42)			> 2 months(*n* = 31)		
	Laparoscopic(*n* = 23)	Open(*n* = 19)	p	Laparoscopic(*n* = 21)	Open(*n* = 10)	p
Sex(Male/Female, n)	4/19	10/9	0.016	1/20	2/8	0.237
Age at surgery (d, M[p25,p75])	41.0[30.0,48.0]	32.0[14.0,42.0]	0.079	98.0[75.5,116.5]	120.0[110.0,138.9]	0.554
Weight at surgery (kg, M[p25,p75])	4.40[3.50,4.70]	4.00[3.60,4.40]	0.456	6.90[6.00,8.46]	7.60[6.00,8.08]	0.512
Preoperative ALT(U/L, M[p25,p75])	19.0[13.0,27.0]	15.0[10.0,16.0]	0.013	45.0[34.0,97.5]	40.5[23.5,68.0]	0.352
Preoperative AST(U/L, M[p25,p75])	32.0[23.0,41.0]	30.0[25.0,48.0]	0.714	55.0[37.0,102.0]	53.0[39.3,94.0]	0.866
Preoperative γ-GT(U/L, M[p25,p75])	229.0[105.0,542.0]	306.0[124.0,665.0]	0.397	49.0[28.5,300.0]	53.0[29.0,521.8]	0.704
Preoperative DBIL(umol/L, M[p25,p75])	13.40[7.55,25.92]	12.60[10.27,20.32]	0.714	4.31[2.17,8.12]	3.61[2.54,26.58]	0.800
Cholestasis before surgery(Yes/No, n)	7/16	6/13	0.936	3/18	3/7	0.358

Mann‒Whitney U tests and Pearson chi-square tests were used. M indicates the median

**Table 4:** Changes in liver function in patients who underwent laparoscopic surgery

Age at surgery	≤ 2 Months (*n* = 23)			> 2 Months (*n* = 21)		
Liver function	At surgery	1 week after surgery	P	At surgery	1 week after surgery	P
ALT(U/L, M[P25,P75])	19.0[13.0,27.0]	29.0[15.0,43.0]	0.014	45.0[34.0,97.5]	31.0[23.5,66.0]	0.035
AST(U/L, M[P25,P75])	32.0[23.0,41.0]	32.0[21.0,45.0]	0.484	55.0[37.0,102.0]	32.0[27.0,62.0]	0.007
γ-GT(U/L, M[P25,P75])	229.0[105.0,542.0]	222.0[120.0,339.0]	0.465	49.0[28.5,300.0]	98.0[49.5,224.5]	0.566
DBIL(umol/L, M[P25,P75])	13.40[7.55,25.92]	6.30[4.80,13.53]	0.001	4.31[2.17,8.12]	2.81[2.40,4.37]	0.013
Cholestasis before surgery(Yes/No, n)	7/16	4/19	0.300	3/18	2/19	1.000

The Wilcoxon test was used to evaluate the ALT, AST, γ-GT, and DBIL levels between the time of surgery and 1 week after surgery at various ages

**Table 5:** Postoperative recovery and complications between the laparoscopic and open surgery groups at various ages

Age at surgery Group	≤ 2 Months (*n* = 42)			> 2 months(*n* = 31)		
	Laparoscopic(*n* = 23)	Open(*n* = 19)	p	Laparoscopic(*n* = 21)	Open(*n* = 10)	p
Operative time(min, M[P25,P75])	340.0[295.0,380.0]	180.0[160.0,205.0]	< 0.001	310.0[257.5,357.0]	200.0[180.0,254.5]	0.005
Anastomotic leakage(Yes/No, n)	1/22	0/19	1.000	0/21	1/9	0.323
Anastomotic stricture(Yes/No, n)	1/22	0/19	1.000	0/21	0/9	-
Initial oral feeding time (d, M[p25,p753])	3.0[2.0,4.0]	5.0[3.0,6.0]	0.042	2.0[2.0,3.0]	4.0[4.0,5.0]	0.002
PN time (d, M[p25,p753])	6.0[5.0,8.0]	8.0[5.0,10.0]	0.094	6.0[5.0,6.5]	8.0[6.8,9.8]	0.007
Postoperative hospital stay(d, M[p25,p753])	7.0[6.0,9.0]	9.0[6.0,11.0]	0.093	7.0[6.0,7.5]	9.0[7.8,10.8]	0.007

Mann‒Whitney U tests and Fisher tests were used

**Table 6:** Postoperative recovery and complications occurred in patients who underwent laparoscopic-assisted surgery at ≤ 2 months and > 2 months of age

Surgery	Laparoscopic assisted surgery		
Age at surgery	≤ 2 Months (*n* = 23)	> 2 months (*n* = 21)	p
Operative time(min, M[P25,P75])	340.0[295.0,380.0]	310.0[257.5,357.0]	0.071
Anastomotic leakage(Yes/No, n)	1/22	0/21	1.000
Anastomotic stricture(Yes/No, n)	1/22	0/21	1.000
Initial oral feeding time (d, M[p25,p753])	3.0[2.0,4.0]	2.0[2.0,3.0]	0.117
PN time (d, M[p25,p753])	6.0[5.0,8.0]	6.0[5.0,6.5]	0.530
Postoperative hospital stay(d, M[p25,p753])	7.0[6.0,9.0]	7.0[6.0,7.5]	0.530

The Mann‒Whitney U test and Fisher test were used

## Discussion

### The proper timing for CC excision in prenatally diagnosed CC infants

In our study, the incidence of cholestasis in CC patients was compared with that in healthy controls at various ages, and nearly 20-40% of the patients developed biliary obstruction, which was more obvious in neonates and at 1–2 months of age. This result indicated that prenatally diagnosed CC patients may be born with no symptoms; however, there is a high risk of biliary obstruction, which can be more obvious at less than 2 months after birth. Except for cholestasis, the total occurrence of clinical symptoms was 28.8%, and approximately 70% of clinical symptoms occurred less than 2 months after birth in our study. Nearly 50% of the symptoms associated with CC are reported to occur in the first 3 months of life [13], and the most common symptom in neonates and young infants is biliary obstruction [15], which may cause hepatic damage and liver fibrosis.

In our study, the CC size was larger in symptomatic patients than in asymptomatic patients with prenatally diagnosed CC, which may indicate that a larger CC tends to cause easier clinical symptoms and earlier surgery. One study reported that a cyst > 5.2 cm in length and > 4.1 cm in width suggested that clinical symptoms might appear and that surgery should be carried out as soon as possible, even during the neonatal period [13]. However, in the present study, no differences in the occurrence of clinical symptoms or CC size were detected at various ages, which indicated that other indices should be detected to explore the timing of CC excision in patients with prenatally diagnosed CC.

In the present study, liver function, represented by ALT and AST levels, was abnormal 2 months after birth in prenatally diagnosed CC patients, which proves that hepatic damage is obvious after 2 months of age. ALT and AST are the most common indices of liver function and are associated with the degree of hepatic cell damage [16, 17]. For CC patients, abnormal ALT and AST levels may be attributed to continued hepatic damage. The incidence of choletasis was significantly greater in CC patients than in controls aged less than 2 months, which may indicate that biliary obstruction leads to continued hepatic cell damage and ultimately results in increased ALT and AST levels. It is necessary to interrupt this continuous hepatic damage by performing CC excision when the patient is less than 2 months old.

The APRI is a popular index for evaluating the degree of liver fibrosis in patients with various hepatic diseases [18–20]. In our study, the APRI was used to evaluate hepatic damage and liver fibrosis, and significantly greater APRIs were found in CC patients than in healthy infants after 2 months of age. This increasing trend in the APRI was consistent with the increasing trends in ALT and AST levels, which confirmed that obvious hepatic damage was prevalent 2 months after birth. In the present study, 73 CC patients were divided into 4 sub-age groups, and 85 relatively normal controls were recruited to evaluate changes in liver function and the APRI, which could reveal early hepatic damage. In 2022, it was reported that surgical treatment is advantageous for patients who are prenatally diagnosed with CC before symptoms appear [13]. According to the findings of our study, patients with prenatally diagnosed CC should receive CC excision within at least 2 months after birth.

### Feasibility of minimally invasive surgery in young prenatally diagnosed CC infants (younger than 2 months)

In our study, 42 patients with prenatally diagnosed CC aged ≤ 2 months and 31 aged > 2 months were further enrolled in the laparoscopic and open groups, respectively. The values of ALT, AST and DBIL significantly decreased from before the operation to 1 week after laparoscopic CC excision at > 2 months of age; however, only DBIL obviously improved at ≤ 2 months. Reports have suggested that delaying surgery by more than 6 months and weighing 5.6 kg may yield better results [8]. Japanese clinical practice guidelines for congenital biliary dilatation [21] also recommend that symptomatic neonates and infants be operated on as soon as possible, whereas elective operations at approximately 3–6 months of age may be considered for asymptomatic patients while liver functions, etc., are monitored carefully. In our study, the ALT and AST levels were abnormal after 2 months of age. Sudden progression toward liver failure, and intracranial hemorrhage attributed to abnormal liver function was reported in neonates and infants with CC [2, 12, 22, 23]. In patients who underwent laparoscopic CC excision within 2 months, DBIL levels obviously decreased 1 week after surgery, indicating that performing laparoscopic surgery in young infants aged less than 2 months was also a feasible option. The reason for the lack of obvious decreases in ALT and AST levels in patients who underwent surgery at less than 2 months of age was that ALT and AST levels were actually normal before surgery at less than 2 months. Moreover, performing laparoscopic surgery in patients less than 2 months old is considered effective compared with performing laparoscopic surgery more than 2 months after birth.

In the present study, the initial oral feeding time was earlier in CC patients who underwent laparoscopic surgery than in CC patients who underwent open surgery at either ≤ 2 months or > 2 months of age. However, the PN length and postoperative stay were shorter in patients who underwent laparoscopic surgery than in patients who underwent open surgery at > 2 months of age. Both of these findings strongly prove the superiority of laparoscopic surgery in CC patients, which is consistent with the findings of previous studies [4, 24]. Laparoscopic surgery is an actual minimally invasive operation and has been proven to be the most important component for enhancing recovery after surgery [25]. Laparoscopic CC excision takes more time than open surgery for CC; however, laparoscopic surgery reduces the exposure of the intestine and the body’s stress reaction [26].

In our study, no higher rate of mid- to long-term anastomotic stricture was found in the laparoscopic group than in the open group at either ≤ 2 months or > 2 months of age after 1–10 years follow-up. According to these findings, the authors suggest that performing laparoscopic surgery in infants with CC is safe; however, a recent meta-review also showed that laparoscopic surgery does not increase the risk of anatomical stricture [27]. In our study, the recovery and complication rates in CC patients aged less than 2 months who underwent laparoscopic surgery were equal to those in CC patients aged more than 2 months who underwent laparoscopic surgery, which confirmed the feasibility of laparoscopic surgery in neonates and young infants (less than 2 months) with prenatally diagnosed CC.

## Conclusion and limitations

Most clinical symptoms attributed to CC occur less than 2 months after birth, while liver function and liver fibrosis may deteriorate after 2 months of age in patients with prenatally diagnosed CC. Laparoscopic surgery for CC in newborns and young infants (either less than or more than 2 months old) is safe and feasible and can shorten the initial oral feeding time without increasing complications such as postoperative anastomotic leakage or stricture. Thus, performing laparoscopic CC excisions within 2 months after birth in patients with prenatally diagnosed CC may be appropriate.

This was a retrospective study in which patients who underwent laparoscopic or open surgery could not be randomized. However, data on continuous postoperative changes in liver function (more than 1 week after surgery) were not available between the laparoscopic group and the open group, and the number of patients was relatively limited. As a result of these limitations, a multicenter study with more detailed clinical features of a large number of patients with prenatally diagnosed CC is recommended to obtain reliable results.

## Data Availability

All the data generated or analyzed during this study are available from the corresponding authors upon reasonable request.

## References

[1] Cazares J, Koga H, Yamataka A (2023). Choledochal cyst. Pediatr Surg Int.

[2] Scalise PN, Yang A, Neumeyer C (2021). Prenatal diagnosis of rapidly enlarging choledochal cyst with gastric outlet obstruction. J Surg Case Rep.

[3] Kowalski A, Kowalewski G, Kalicinski P, et al. Choledochal Cyst Excision in Infants-A Retrospective Study. Children-Basel. 2023;10(2). 10.3390/children10020373. 10.3390/children10020373 PMC9954880 36832502

[4] Zhang X, Jin J, Qiu T (2023). The strategy of laparoscopic surgery for asymptomatic antenatally diagnosed choledochal cyst. Bmc Surg.

[5] Cook KM, De Asis-Cruz J, Kim JH (2023). Experience of early-life pain in premature infants is associated with atypical cerebellar development and later neurodevelopmental deficits. Bmc Med.

[6] Lee JH, Zhang J, Wei L (2015). Neurodevelopmental implications of the general anesthesia in neonate and infants. Exp Neurol.

[7] Tanaka H, Sasaki H, Wada M (2015). Postnatal management of prenatally diagnosed biliary cystic malformation. J Pediatr Surg.

[8] van den Eijnden M, de Kleine RH, de Blaauw I (2017). The timing of surgery of antenatally diagnosed choledochal malformations: a descriptive analysis of a 26-year nationwide cohort. J Pediatr Surg.

[9] Chen S, Yin T, Li L (2023). Correlation of ectopic distal location of papilla of Vater and clinical characteristics in pediatric choledochal cysts. Pediatr Surg Int.

[10] Okada T, Honda S, Miyagi H (2013). Liver fibrosis in prenatally diagnosed choledochal cysts. J Pediatr Gastr Nutr.

[11] Diao M, Li L, Cheng W (2012). Timing of surgery for prenatally diagnosed asymptomatic choledochal cysts: a prospective randomized study. J Pediatr Surg.

[12] Singh RJ, Ali MM, Rashi R (2023). Giant choledochal cyst in infant: a rare case report. Afr J Paediatr Surg.

[13] Guan X, Li J, Wang Z (2022). Timing of operation in children with a prenatal diagnosis of choledochal cyst: a single-center retrospective study. J Hepato-Bil-Pan Sci.

[14] Fawaz R, Baumann U, Ekong U (2017). Guideline for the Evaluation of Cholestatic Jaundice in infants: joint recommendations of the North American Society for Pediatric Gastroenterology, Hepatology, and Nutrition and the European Society for Pediatric Gastroenterology, Hepatology, and Nutrition. J Pediatr Gastr Nutr.

[15] Farooq MA, Khan SA, Malik MI (2023). Choledochal Cyst in Children, presentation and outcome – 10 years’ experience from a tertiary care center in Pakistan. Pak J Med Sci.

[16] Pinheiro B, Rodrigues JG, Dias F (2023). Hepatic damage caused by flaviviruses: a systematic review. Life Sci.

[17] Ojeaburu SI, Oriakhi K (2021). Hepatoprotective, antioxidant and, anti-inflammatory potentials of gallic acid in carbon tetrachloride-induced hepatic damage in Wistar rats. Toxicol Rep.

[18] Lyu H, Ye Y, Wang B (2023). FIB-4 and APRI scores for progressive liver fibrosis diagnosis in children with biliary atresia. Front Pediatr.

[19] Moosavy SH, Eftekhar E, Davoodian P (2023). AST/ALT ratio, APRI, and FIB-4 compared to FibroScan for the assessment of liver fibrosis in patients with chronic hepatitis B in Bandar Abbas, Hormozgan, Iran. Bmc Gastroenterol.

[20] Liu K, Qin M, Tao K (2021). Identification and external validation of the optimal FIB-4 and APRI thresholds for ruling in chronic hepatitis B related liver fibrosis in tertiary care settings. J Clin Lab Anal.

[21] Ishibashi H, Shimada M, Kamisawa T (2017). Japanese clinical practice guidelines for congenital biliary dilatation. J Hepato-Bil-Pan Sci.

[22] Grover SB, Malhotra S, Pandey S et al. Erratum to Imaging diagnosis of a giant choledochal cyst in an infant [Radiology Case Reports 17 (2022) 404–411]. Radiol Case Rep 2023, 18(2):735.10.1016/j.radcr.2022.10.095. 10.1016/j.radcr.2021.10.051 PMC8649116 34925674

[23] Shrestha AL, Mishra A (2023). Infantile choledochal cyst presenting with an epigastric bilioma: an iceberg phenomenon. Int J Surg Case Rep.

[24] Murakami M, Kaji T, Nagano A (2021). Complete laparoscopic choledochal cyst excision and hepaticojejunostomy with laparoscopic Roux-Y reconstruction using a 5-mm stapler: a case of a 2-month-old infant. Asian J Endosc Surg.

[25] Arena S, Di Fabrizio D, Impellizzeri P (2021). Enhanced recovery after gastrointestinal surgery (ERAS) in Pediatric patients: a systematic review and Meta-analysis. J Gastrointest Surg.

[26] Yeh A, Butler G, Strotmeyer S (2020). ERAS protocol for pediatric laparoscopic cholecystectomy promotes safe and early discharge. J Pediatr Surg.

[27] Tanaka R, Nakamura H, Yoshimoto S (2022). Postoperative anastomotic stricture following excision of choledochal cyst: a systematic review and meta-analysis. Pediatr Surg Int.

